# Screening for renal impairment in viral hepatopathy B: it is time to begin

**DOI:** 10.2144/fsoa-2023-0019

**Published:** 2023-07-06

**Authors:** Mona Boudabbous, Fatma Bouzid, Ikram Agrebi, Héla Gdoura, Lassad Chtourou, Manel Moalla, Leila Mnif, Ali Amouri, Khaoula Kammoun, N Tahri

**Affiliations:** 1 Gastroenterology Department, Hédi Chaker Hospital, Sfax, Tunisia; 2 Nephrology Department, Hédi Chaker Hospital, Sfax, Tunisia; 3 Department medecine B, Medecin Sfax University, Sfax university, Sfax, Tunisia

**Keywords:** albuminuria, hepatitis B, renal failure

## Abstract

**Aim:**

The aim of this study was to assess the value of routine screening for renal damage in cases of B viral infection, by looking for proteinuria and elevated creatinemia.

**Materials & methods:**

We investigate the frequency and associated factors of renal impairment in patients with viral B hepatopathy.

**Results:**

Pathological albuminuria was confirmed in 44.73%. The chronic kidney disease with slightly decreased filtration rate was found in 21.05%. In multivariate analysis, only BMI was an independent factor for pathological albuminuria (p = 0.013) and only age was an independent predictor of chronic kidney disease (p = 0.056).

**Conclusion:**

Urine dipstick and creatinemia are useful for routine screening in viral B hepatopathy especially in the elderly and overweight.

The liver and the kidneys have an important function in the metabolism and the homeostasis of the body. They have complex interactions [1]. Therefore, hepatopathies, in particular viral hepatitis B, can have a direct or indirect impact on kidneys physiology and function [2]. The first cases of renal damage in cirrhotic patients were reported in 1863 [3]. Gradually, the renal involvement occurring during viral B liver disease was highlighted. In order to avoid the discovery of an advanced chronic kidney disease (CKD) with the burden of hemodialysis, a simple screening for markers of the renal involvement in patients with B viral hepatopathy allows its management at an early stage. As a result, it delays its evolution, improves its prognosis and consequently upgrades the patient’s life expectancy and quality.

It is well-known that HBV infection is related to immune-complex mediated glomerulonephritis. In addition to glomerulonephritis, several studies found HBV infection was associated with an increased risk of CKD in cross-sectional and longitudinal studies. The decline in renal function observed in chronic hepatitis B can be life-threatening if not diagnosed and managed. However, it is difficult to identify vulnerable patients who may have renal dysfunction, as the relationship between the HBV and the kidneys is complex [4]. Indeed, on the one hand, in the history of the disease, the presence of immunological mechanisms involving viral antigens and specific anti-HBV antibodies would be responsible for several extrahepatic manifestations. Among these manifestations, HBV glomerulonephritis (HBV-GN) and periarteritis nodosa (PAN) are the most characteristic and likely to induce renal lesions [5]. Prolonged treatment with tenofovir disoproxil fumarate, on the other hand, has been associated with rare cases of proximal renal tubular dysfunction, usually occurring after months or years of treatment. This renal injury is most often manifested as a Fanconi syndrome with acute renal failure and distal tubular damage in the form of nephrogenic diabetes insipidus [6].

Despite all these data, to date, there are no national or international recommendations for the systematic screening of renal dysfunction in viral B hepatopathy. In this framework, the aim of this study was to assess the value of routine screening for renal damage in cases of B viral infection, by looking for proteinuria and elevated creatinemia. Thus, we conducted this study to investigate the frequency and associated factors of renal impairment in patients with viral B hepatopathy.

## Materials & methods

This was a cross-sectional study including patients followed for viral B hepatopathy at the gastroenterology department, who had consulted from 1 October 2020 to 31 December 2020.

As the aim was to assess the value of screening for renal impairment in patients with no other reason for monitoring renal function, we excluded patients with diabetes, hypertension or systemic diseases that could cause renal failure (e.g., lupus, connectivites…) and previously diagnosed nephropathy (whatever its mechanism).

The determination of renal function was based on the calculation of the estimated glomerular filtration rate (GFR) from the creatinemia using the simplified modification of diet in renal disease (MDRD) formula via the Medicalcul software available on the Android mobile platform (http://medicalcul.free.fr/mdrdsimp.html)

The diagnosis of CKD was made when there was a GFR that had been impaired for more than 3 months (if available) or after eliminating any cause of acute renal failure with persistent impaired renal function during follow-up.

– CKD with a slightly decreased GFR was considered in case of an estimated GFR between 60 and 90 ml/min/1.73 m^2^.

– Renal failure was defined as GFR <60 ml/min/1.73 m^2^ [7].

According to the literature, the interpretation of the fibroscan value varies according to the etiology of the liver disease [8,9]

For chronic hepatitis B:

- If the alanine aminotransferases (ALT) level is normal, we have defined:
  -  Absence of fibrosis or minimal fibrosis (F0F1) when liver elasticity is less than 6 kPa
  - Presence of fibrosis (F2 score) between 6 and 9 kPa
  -  Severe fibrosis (scores ≥F3) or even cirrhosis for a value above 9 kPa
- If ALT is elevated but less than five-times normal, we defined:
  -  Absence of fibrosis or minimal fibrosis (F0F1) when liver elasticity is less than 6 kPa;
  - Presence of fibrosis (F2 score) between 6 and 12 kPa
  -  Severe fibrosis (scores ≥F3) or even cirrhosis for a value above 12 kPa

All the patients had a urine dipstick examination (UD), a blood test including: creatinemia, urea, fasting blood glucose, transaminases, alkaline phosphatase, gamma glutamyl transferase; bilirubinemia, lipid profile; PT, PEE and CBC, abdominal ultrasound and liver elastography.

Proteinuria and/or hematuria and/or nitrituria and/or leukocyturia on the UD is/were considered positive for a result above one cross

Patients with a suspected urinary tract infection (UTI) on UD screening (positive leukocyturia and/or nitrituria) had undergone a cyto bacteriological examination of the urine. Then a UD examination was repeated after treatment of the confirmed UTI.

Patients with positive proteinuria on UD had a quantitative determination of proteinuria and creatininuria in urine sample in order to calculate the albuminuria/urine creatinine ratio.

Patients with positive hematuria on UD had a red blood cells-leukocytes-minute (BLM). Patients with confirmed microalbuminuria (albuminuria/urine creatinine ratio between 3 and 30 mg/mmol, or 30 and 300 mg/g) or proteinuria (albuminuria/urine creatinine ratio >30 mg/mmol or >300 mg/g) and/or CKD with decreased GFR (GFR <90 ml/min/1.73 m^2^ by MDRD) are referred to the nephrology department for more investigation and management.

The statistical analysis of the data was performed with the statistical software Statistical Package for Social Sciences.

In a first step, we performed a descriptive study of the characteristics of the patients, of the viral hepatopathy B and of the frequency of the stigmata of the renal damage.

Second, we conducted a uni and multivariate analytical study to look for dependent and independent factors predictive of renal damage in all patients with viral hepatopathy B.

We carried out a univariate analysis based on the following tests:

-  For qualitative variables: percentage comparisons were made using the Chi-square test when the conditions for application were met, and the Fisher test when not.
-  For quantitative factors: When the response variable was a 2-modality qualitative variable, the comparison of means on unpaired series was carried out using the Student’s *t*-test when the normality of the distribution was verified, and using the nonparametric Mann–Whitney–Wilcoxon test for unpaired series when this was not verified.

Correlations between two quantitative variables were tested using Pearson’s test when the conditions were met, and Spearman’s test when they were not.

A multivariate analysis was performed using a conditional logistic regression model including variables where the p was ≤0.05 at the end of the univariate analysis, in addition to factors found in the literature. A statistical significance level of 0.1 was retained, with the adoption of the best models allowing a higher percentage of variables to be well classified in the univariate analysis.

In all statistical tests, the significance level was 0.05 (p < 0.05). In multivariate analysis, the significance level was 0.1 (p < 0.1).

## Results

Our population included 38 patients. Among them, there were 29 men (76.3%). The average age was 53.5 ± 10 years with extremes of 33 and 74 years. Thirteen patients (34.2%) had a family history of viral hepatopathy B and three patients (7.9%) had a family history of nephropathy. HBeAg was negative for 29 patients (76.3%). Viral load was above 20,000 IU/ml in 19 of 35 patients (54.3%) initially and only in one of 30 patients (3.3%) currently. It became negative in 16 treated patients. Due to the unavailability of other antiviral molecules, all our patients were treated with entecavir. Among 27 patients on antiviral treatment (71%), 24 patients (88.88%) had good therapeutic observance. About one third of the patients had liver cirrhosis (n = 13, 34.2%). Thirteen patients (34.2%) were overweight and nine patients (23.7%) were obese with a mean BMI of 26.25 ± 4.13 kg/m^2^. Ten patients (13.15%) had dyslipidemia. A total of 30 patients (79%) had undergone oesogastroduodenal fibroscopy. Among them, 15 patients (50%) had esophageal varices and nine patients (30%) had hypertensive gastropathy. During UD screening, 21 patients (55.3%) had proteinuria and seven patients (18.4%) had hematuria. We confirmed the pathological albuminuria in 17 patients (44,73%). The CKD with slightly decreased filtration rate was found in eight patients (21,05%).

Among 28 patients with viral B liver disease, seven patients (25%) had moderate liver fibrosis and 12 patients (42.9%) had liver cirrhosis. While the remaining nine patients (23.7%) had normal fibroscan values indicating the absence of fibrosis (Table 1).

**Table 1.** Descriptive analysis of our patients.

Features of our population		n (%)
Patients (n)		38
Sex	Male	29 (76.3)
	Female	9 (23.7)
Age (years)		53,5 ± 10
Family history of viral hepatopathy B		13 (34.2)
Family history of nephropathy		3 (7.9%)
HBe Ag status	Negative	29 (76.3)
	Slightly positive	3 (7.9)
	Positive	3 (7.9)
Viral load >20,000 IU/ml	Initially	19 (54.3)
	Currently	1 (3.3)
Patients on antiviral treatment		27 (71%)
Patients on a good therapeutic observance		24 (88.88)
Liver cirrhosis		13 (34.2)
Decompensated cirrhosis		9 (23.7)
Hepatocellular carcinoma		3 (7.8)
Smoking		23 (60.52)
Drinking alcohol		15 (39.47)
Weight status	Overweight	13 (34.2)
	Obesity	9 (23.7)
Dyslipidemia		10 (13.15)
Data of abdominopelvic ultrasound scan	Liver dysmorphia	14 (38.88)
	Signs of portal hypertension	23 (63.73)
Data of oesogastroduodenal fibroscopy	Esophageal varices	15 (50)
	Hypertensive gastropathy	9 (30)
Data of liver fibroscan	Moderate liver fibrosis	7 (25)
	liver cirrhosis	12 (42.9)
	Absence of fibrosis	9 (23.7)
Urine dipstick screening	Proteinuria	21 (55.3)
	Hematuria	7 (18.4)
Pathological albuminuria	Microalbuminuria	15 (39.47)
	Proteinuria	2 (5.26)
Chronic kidney disease with slightly decreased filtration rate		8 (21.05)

In univariate analysis, only hematuria on UD was a predictive factor for pathological albuminuria (p = 0.018). However, none of the biological, radiological and the histological data nor the fibroscan value were associated with the presence of pathological albuminuria. None of all the data was associated with CKD (Tables 2 & 3).

**Table 2.** Factors associated with the presence of pathological albuminuria in univariate analysis.

Factors		Pathological albuminuria	Absence of microalbuminuria	p-value
Age (years)		54	53	0.82
Sex	Male, n (%)	14 (82.4)	10 (62.5)	0.62
	Female, n (%)	3 (17.6)	6 (37.5)	
Smoking, n (%)		11 (64.7)	8 (50)	0.39
BMI >25 kg/m^2^		8 (50)	11 (68.8)	0.28
Hematuria		6 (35.3)	0	**0.018**
Hypercholesterolemia		3 (21.4)	0	0.24

Bold values are statistically significant. For the univariate study, the significance threshold for p is 0.05, for the multivariate study, the significance threshold for p is 0.1.

**Table 3.** Factors associated with the renal function in univariate analysis.

Factors	Chronic kidney disease	Normal renal function	p-value
Age (years)	58.7	51.1	0.057
Smoking, n (%)	4 (57.1)	17 (60.7)	0.59
BMI >25 kg/m^2^, n (%)	5 (71.4)	15 (55.6)	0.38
Hypercholesterolemia, n (%)	0	3 (14.3)	0.51
Portal hypertension (PHT), n (%)	3 (42.9)	11 (39.3)	0.59

In multivariate analysis, only BMI was an independent factor for pathological albuminuria (p = 0.013) and only age was an independent predictor of CKD with a slightly decreased filtration rate (p = 0.056). (Tables 4 & 5).

**Table 4.** Factors associated with the presence of pathological albuminuria in multivariate analysis.

Factors	p-value	OR
Gender	0.26	5
Smoking	1	1
BMI	**0.013**	**0.8**
Hematuria	0.8	1.33

Bold values are statistically significant. For the univariate study, the significance threshold for p is 0.05, for the multivariate study, the significance threshold for p is 0.1.

OR: Odds ratio.

**Table 5.** Factors associated with the chronic kidney disease in multivariate analysis.

Factors	p-value	OR
Age	**0.056**	**1.1**
Sex	0.94	1.1
Smoking	0.95	0.9
BMI >25 kg/m^2^	0.33	1.1

Bold values are statistically significant. For the univariate study, the significance threshold for p is 0.05, for the multivariate study, the significance threshold for p is 0.1.

OR: Odds ratio.

## Discussion

The interest of our study lies in the high frequency of proteinuria and renal insufficiency during viral B hepatopathy, which supports systematic screening by hepatologists for these abnormalities via urine strips and creatinemia measurement. Screening process for hepatitis B renal impairment developed by the study is universal, and the markers and predictors screened are suitable for all hepatitis B patient populations. The screening process for albuminuria by UD is a highly sensitive method for detecting proteinuria, but it frequently presents false positives (alkaline urine [pH >7], concentrated urine [>1025] or contaminated urine [UTI, menstruation]). For these reasons, patients with positive proteinuria on UD had a quantitative determination of proteinuria and creatininuria in urine sample in order to calculate the albuminuria/urine creatinine ratio as recommended [10]

The staging system (which comprises five stages, 1–5) defines CKD on the basis of either evidence of kidney damage (proteinuria, hematuria or anatomical abnormality) or an impaired GFR less than 60 ml/min/1.73 m^2^, present on at least two occasions over 3 months or longer. The adoption of this staging system is recommended. For measurement of renal function, it is recommended for all patients to estimate GFR from serum creatinine using isotope dilution mass spectrometry traceable simplified MDRD equation [11]

The association between hepatitis B virus and the presence of proteinuria on UD has been well studied in the literature. Gupta and Quigg reported that renal involvement in chronic hepatitis B infection is manifested by microalbuminuria or even proteinuria and microscopic hematuria [12]. In accordance with these findings, among our 38 patients with HBV infection, 21 patients (55.3%) had proteinuria on UD. We confirmed the pathological albuminuria in 17 patients (44.73%). A total of 15 patients (39.47%) had microalbuminuria and two patients (5.26%) had proteinuria.

The CKD with slightly decreased filtration rate was found in eight patients (21,05%). According to the University National Hospital of Cotonou, the prevalence of proteinuria on UD was 12.5% in 105 patients followed for chronic hepatitis B [13]. This prevalence is less than the one found in our population. This may be explained by the small sample size in our study.

In fact, the renal damage during chronic hepatitis B can be explained first by the virus and the immunological reactions it has induced and second by the impact of antiviral treatment on the kidneys. Zhang *et al.* reported the presence of HBc, HBe and HBs antigens in the kidney of patients with chronic hepatitis B [14]. He *et al.* reported the existence of HBV DNA in the nucleus and cytoplasm of mesangial cells and glomerular epithelium [15].

Regarding antiviral treatment, a meta-analysis published in 2017 reported that both antiviral molecules tenofovir and entecavir can affect renal function with a higher risk of renal dysfunction and hypophosphatemia in patients on tenofovir compared with entecavir with a statistically significant difference (p = 0.0034 and p = 0.006 respectively) [16]. Another meta-analysis showed that telbivudine, another antiviral molecule, had a protective effect on the kidneys whereas Entecavir was associated with a decline in GFR [17]. Thus, treatment with entecavir could be the cause of renal impairment in our patients with viral B liver disease and a negative viral load. Therefore, a Chinese study published in 2020 recommended screening for renal impairment (hematuria, proteinuria and/or decline in GFR) in patients with chronic hepatitis B and monitoring the progression of renal function in the long term due to the frequency of occurrence of glomerular nephropathy during this viral infection [18]. Screening for markers of renal impairment in chronic HBV infected patients revealed renal dysfunction in 55.26% of our population. This prevalence of renal impairment during chronic HBV infection is close to that found in the literature. Indeed, in France, the prevalence of renal abnormalities during chronic hepatitis B infection was 55.8% in 2012 [19]. However, in China, the prevalence of CKD in patients with hepatitis B was 3.8% after a 5-year follow-up. In this study, in univariate analysis, hepatitis B was associated with GFR decline while it was not associated with pathological albuminuria. In a multivariate study, age was a predictive factor for CKD [20].

In the study of Sehonou *et al.*, renal function decline was significantly associated with age over 50 years (OR: 0.32; p = 0.0125) whereas antiviral treatment was not associated with either renal damage or progression (OR: 0.63; p = 028) [13]. In accordance with these results, in our study, only BMI was predictive of abnormal albuminuria (p = 0.013) and only age was predictive of CKD with slightly decreased filtration rate (p = 0.056), in multivariate analysis.

A prospective German study reported that the decline in GFR is 2 ml/min/1.73 m^2^ per year in 60 untreated HBsAg positive patients after 2 years of follow-up [21]. As the physiological decline in GFR is estimated to be 1 ml/min/1.73 m^2^ per year from the age of 40 years in healthy subjects. This reflects the impact of HBV infection on the acceleration of GFR decline.

Two meta-analyses investigating the relationship between HBV infection and CKD risk showed that HBV exposure was not associated with the development of CKD or proteinuria in Asian and European populations respectively [16,22]. The intervention of other factors such as the B virus genotype, other risk factors for kidney disease, genetic factors, immunological factors, etc. could explain these discordant results.

In contrast with our results, some authors have noted the association between HBeAg and proteinuria [23]. Similarly, a recent study published in 2022 including 94 patients with CKD associated with chronic HBV infection and 548 chronic HBV patients without renal involvement showed that the risk factors for CKD in chronic hepatitis B were HBsAg, HBeAg, dyslipidaemia and duration of infection [24]. The lack of significant association in our study with these factors could be explained by a lack of statistical power due to a relatively small sample size due to COVID epidemic. This was the main limitation of our work

Despite its limitations, our study provides strong arguments in favor of routine screening for renal impairment during B viral infection using UDs and regular monitoring of creatinemia, particularly in obese and elderly subjects.

## Conclusion

In the light of our results and those found in the literature, a UD and creatinine measurement in the case of viral B hepatopathy are useful for screening for markers of renal impairment. In our study, pathological albuminuria and CKD with a slightly decreased GFR were respectively found in 44.73 and 21.05% of our patients. Only BMI was associated with pathological albuminuria and only age was associated with CKD. Thus, simple screening allows for early management and close monitoring of renal damage at an early stage.

**Summary points**

- Our study highlights the potential value of systematic screening for renal failure in patients with B virus, particularly elderly subjects or those with a high BMI.
- We confirmed pathological albuminuria in 44.73% of cases. Chronic kidney disease with a slightly decreased filtration rate was found in over 20% of cases.
- In univariate analysis, only dipstick hematuria was predictive of pathological albuminuria (p = 0.018). However, none of the biological, radiological or histological data, nor the value of the fibroscan, were associated with the presence of pathological albuminuria or renal failure.
- Only BMI was an independent factor in pathological albuminuria (p = 0.013) and only age was an independent predictor of chronic kidney disease with a slightly decreased filtration rate (p = 0.056).

## References

[1] Bonavia A, Singbartl K. Kidney injury and electrolyte abnormalities in liver failure. Semin. Respir. Crit. Care Med. 39(5), 556–565 (2018). 30485886 10.1055/s-0038-1673616

[2] Wong F. Liver and kidney diseases. Clin. Liver Dis. 6(4), 981–1011 (2002). 12516203 10.1016/s1089-3261(02)00053-3

[3] Flint A. Clinical report on hydro-peritoneum, based on an analysis of forty-six cases. Am. J. Med. Sci. 45(90), 306–339 (1863).

[4] Atta MG, Fine DM. Editorial comment: tenofovir nephrotoxicitythe disconnect between clinical trials and real-world practice. AIDS Read. 19, 118–119 (2009). 19334329

[5] Terrier Benjamin, Cacoub Patrice. Virus de l'hépatite B, manifestations extrahépatiques immunologiques et risque de réactivation virale. Rev. Med Int. 32(10), 622–627 (2011). 10.1016/j.revmed.2010.08.013 20870315

[6] Verhelst D, Monge M, Meynard JL, Fouqueray B, Mougenot B, Girard PM. Fanconi syndrome and renal failure induced by tenofovir: a first case report. Am. J. Kidney Dis. 40, 13311333 (2002). 10.1053/ajkd.2002.36924 12460055

[7] Nathalie P. Guide du parcours de soins – Maladie rénale chronique de l'adulte (MRC). 85 Haute Autorité de santé – 2021 – ISBN : 978-2-11-162629-4 (2021).

[8] European Association for Study of Liver, Association Latino Americana para el Estudio del Higado. EASL-ALEH Clinical Practice Guidelines: non-invasive tests for evaluation of liver disease severity and prognosis. J. Hepatol. 63(1), 237–264 (2015). 25911335 10.1016/j.jhep.2015.04.006

[9] European Association for the Study of the Liver. Clinical Practice Guidelines on non-invasive tests for evaluation of liver disease severity and prognosis - 2021 update. J. Hepatol. 75(3), 659–689 (2021). 34166721 10.1016/j.jhep.2021.05.025

[10] Haute Autorité de Santé / Service Évaluation des actes professionnels. Rapport albuminurie/creatininurie- Rapport d'évaluation. (2011). www.has-sante.fr/upload/docs/application/pdf/2011-12/rapport__albuminurie_creatininurie_2011-12-27_14-57-31_440.pdf

[11] Groupe de travail de la Société de néphrologie. Évaluation de la fonction rénale et de la protéinurie pour le diagnostic de la maladie rénale chronique chez l'adulte. Recommandations pour la pratique clinique. Nephrol. Ther. 5(4), 302–305 (2009). 19406695 10.1016/j.nephro.2009.02.011

[12] European Association For The Study Of The Liver. EASL clinical practice guidelines: management of chronic hepatitis B virus infection. J. Hepatol. 57(1), 167–185 (2012). 22436845 10.1016/j.jhep.2012.02.010

[13] Séhonou J, Kpossou AR, Amanda TO, Sokpon CNM, Vignon RK, Vigan J. Hépatite virale B et insuffisance rénale: prévalence et facteurs associés au Centre National Hospitalier et Universitaire de Cotonou. Pan. Afr. Med. J. 31, 121 (2018). 31037181 10.11604/pamj.2018.31.121.16498 PMC6462369

[14] Zhang JH, Li LS, Zhou H. Is there a hepatitis B virus-associated glomerulonephritis? Identification of HBsAG, HBcAG and HBeAG in kidney with monoclonal antibodies. Chin. Med. J. 102(7), 496–504 (1989). 2517065

[15] He XY, Fang LJ, Zhang YE, Sheng FY, Zhang XR, Guo MY. *In situ* hybridization of hepatitis B DNA in hepatitis B-associated glomerulonephritis. Pediatr. Nephrol. Berl. Ger. 12(2), 117–120 (1998). 10.1007/s004670050417 9543368

[16] Fabrizi F, Donato FM, Messa P. Association between hepatitis B virus and chronic kidney disease: a systematic review and meta-analysis. Ann. Hepatol. 16(1), 21–47 (2017). 28051791 10.5604/16652681.1226813

[17] Tsai MC, Chen CH, Tseng PL Does Nucleos(t)ide analogues treatment affect renal function in chronic hepatitis B patients who have already decreased eGFR? A Longitudinal Study. PLOS ONE 11(3), e0149761 (2016). 26964034 10.1371/journal.pone.0149761 PMC4786133

[18] Liu Y, Shi C, Fan J, Wang B, Li G. Hepatitis B-related glomerulonephritis and optimization of treatment. Expert. Rev. Gastroenterol. Hepatol. 14(2), 113–125 (2020). 31951758 10.1080/17474124.2020.1717948

[19] Amet S, Launay-Vacher V, Pol S Prévalence des anomalies rénales dans l'hépatite B chronique: résultats de l'étude HARPE. Néphrologie. Thérapeutique. 8(5), 406–407 (2012).

[20] Kong XL, Ma XJ, Su H, Xu DM. Relationship between occult hepatitis B virus infection and chronic kidney disease in a Chinese population-based cohort. Chronic Dis. Transl. Med. 2(1), 55–60 (2016). 29063026 10.1016/j.cdtm.2016.07.001 PMC5643595

[21] Cai J, Fan X, Mou L Association of reduced renal function with hepatitis B virus infection and elevated alanine aminotransferase. Clin. J. Am. Soc. Nephrol. 7(10), 1561–1566 (2012). 22859746 10.2215/CJN.07410711 PMC3463211

[22] Cai QC, Zhao SQ, Shi TD, Ren H. Relationship between hepatitis B virus infection and chronic kidney disease in Asian populations: a meta-analysis. Ren. Fail. 38(10), 1581–1588 (2016). 27756165 10.1080/0886022X.2016.1229548

[23] Fabrizi F, Cerutti R, Ridruejo E. Hepatitis B virus infection as a risk factor for chronic kidney disease. Expert. Rev. Clin. Pharmacol. 12(9), 867–874 (2019). 31456441 10.1080/17512433.2019.1657828

[24] Liu Y, Wang X, Xu F Risk factors of chronic kidney disease in chronic hepatitis b: a hospital-based case–control study from China. J. Clin. Transl. Hepatol. 10(2), 238–246 (2022). 35528983 10.14218/JCTH.2021.00082 PMC9039709

